# Germline variants detected by multigene panel testing in patients with suspected hereditary breast cancer

**DOI:** 10.1007/s00595-025-02994-3

**Published:** 2025-01-20

**Authors:** Yusa Togashi, Masayuki Nagahashi, Mina Kashima, Chiho Okada, Chinatsu Kinjo, Ayako Miyazaki, Mako Ueda, Hiroshi Tsubamoto, Hideaki Sawai, Yasuo Miyoshi

**Affiliations:** 1 Department of Surgery, Division of Breast and Endocrine Surgery, School of Medicine, Hyogo Medical University, 1-1 Mukogawa-cho, Nishinomiya, Hyogo 663-8501 Japan; 2 Department of Clinical Genetics, Hyogo Medical University, 1-1 Mukogawa-cho, Nishinomiya, Hyogo 663-8501 Japan; 3 Department of Obstetrics and Gynecology, Hyogo Medical University, 1-1 Mukogawa-cho, Nishinomiya, Hyogo 663-8501 Japan

**Keywords:** Hereditary breast cancer, Multigene panel testing, Next-generation sequencing, Variant of uncertain significance, Japanese

## Abstract

**Purpose:**

To clarify the status of multigene panel testing for suspected hereditary breast cancer in our institute, and disclose the characteristics of the variants detected.

**Methods:**

This was a retrospective study of individuals who underwent next-generation sequencing-based multigene panel testing at our institute to investigate hereditary genetic variants for suspected hereditary breast cancer.

**Results:**

We identified 36 women who underwent multigene panel testing: 8 (22.2%) had a pathogenic variant, with or without other variants of uncertain significance (VUSs); 15 (41.7%) had VUSs only; and 13 (36.1%) had negative genetic test results. Of the eight pathogenic variants, five were *BRCA2* variants and one each were *BRCA1*, *MLH1,* and *RINT1* variants. The VUSs included *BRCA1* and *BRCA2*, as well as other breast cancer-associated genes, such as *ATM*, *CDH1*, *CHEK2*, and *PALB2*. Referring to the latest ClinVar database, one of the variants identified as a VUS at diagnosis was re-determined as likely pathogenic, and three of the variants identified as VUSs at diagnosis were re-determined as benign.

**Conclusion:**

VUSs are frequently identified during testing and it is important to monitor these individuals because VUS evaluations can change over time.

**Supplementary Information:**

The online version contains supplementary material available at 10.1007/s00595-025-02994-3.

## Introduction

Breast cancer is the most common cancer in Japanese women [1, 2], and approximately 10% of these cancers are thought to be hereditary, with 4%–5% (about half of hereditary breast cancers) reported to be caused by *BRCA1* or *BRCA2* variants [3–5]. The usefulness of prophylactic surgery and surveillance for hereditary breast cancer in breast cancer patients with germline *BRCA1* or *BRCA2* variants has been demonstrated [6]. Moreover, germline testing for *BRCA1* or *BRCA2* to diagnose hereditary breast and ovarian cancer syndrome has been covered by National Health Insurance in Japan since 2020 [7]. In addition to *BRCA1* and *BRCA2*, several other genes are associated with an increased risk of the development of breast cancer, including *PALB2*, *TP53*, *PTEN*, *CDH1*, *ATM*, *and CHEK2* [8–10]. For example, the estimated absolute lifetime risk of breast cancer associated with *PALB2* variants is about 40%: a high frequency that approaches the risk of breast cancer in individuals with *BRCA2* variants [10]. The United States and European countries are also focusing their efforts on the diagnosis, surveillance, and prevention of hereditary breast cancer caused by variants in genes other than *BRCA1/2*. In fact, the National Comprehensive Cancer Network and other guidelines include recommendations for individuals with those variants [11–13].

Recently, next-generation sequencing (NGS) has enabled the multigene panel testing of these variants, providing information about multiple gene variants at the same time and at a low cost [14–17]. Multigene panel testing can examine the causative genes of hereditary tumors other than *BRCA1/2* efficiently in patients with suspected hereditary breast cancer but who have negative *BRCA1/2* genetic test results. Moreover, patients with suspected hereditary tumors may have not only one, but multiple causative genes for hereditary tumors and multigene panel testing may be useful. Although germline testing for *BRCA1*/*2* has become widespread in Japan [18], multigene panel testing for germline variants is conducted in a limited number of facilities [19, 20]. Multigene panel testing is not yet widely adopted in Japan as it is not covered by insurance, so the patient must pay the full cost of the test. In addition, for those found to have variants by the multigene panel test, there is no National Health Insurance coverage for prophylactic resection and the medical system does not yet provide adequate surveillance for these individuals.

As approximately half of the causative genes for hereditary breast cancer are genes other than *BRCA1/2*, multigene panel testing may be beneficial, especially when there is a strong family history of the disease or when the disease occurs at a young age and a *BRCA1/2* gene variant is absent [19, 20]. Our institution introduced multigene panel testing in 2016 for patients with suspected hereditary breast cancer. The aim of this study was to clarify the current status of multigene panel testing performed for suspected hereditary breast cancer in our institute, and to disclose the characteristics of the variants detected in this setting.

## Methods

### Study participants

The subjects of this retrospective study were women who underwent multigene panel testing using NGS at Hyogo Medical University Hospital between February, 2016 and September, 2024 to investigate hereditary genetic variants for suspected hereditary breast cancer. The selection criteria were patients with suspected hereditary breast cancer based on a comprehensive genomic profiling test for recurrent breast cancer, or patients with suspected hereditary breast cancer based on their own history of breast cancer and/or family history. The Institutional Review Board of the Hyogo College of Medicine approved this study (No. 0449), which was planned in accordance with the Declaration of Helsinki. Written informed consent for participation in this study was obtained from all patients involved.

### NGS-based multigene panel testing

Our institution has several multigene panel tests available and we presented the client with a choice of tests that could cover the genes for their expected familial tumor and then selected the test according to the patient’s preferences after a thorough discussion during pre-test genetic counseling, as described later. The larger 160-gene panel test was prepared as an option to meet the needs of patients who wanted to conduct a broad search. Specifically, each participant underwent one of the following tests to detect germline variants in multiple genes: Invitae Multi-Cancer Panel (84 genes; Invitae, San Francisco, CA, USA); Sentis Hereditary Cancer Panel for women (74 genes; BGI, Cambridge, MA, USA); Sentis Hereditary Breast and Ovarian Cancer (26 genes; BGI); and Comprehensive Hereditary Cancer Panel Plus (160 genes; Blueprint Genetics, Espoo, Finland). Blood samples were collected and processed according to the manufacturers’ instructions. Supplementary Table 1 lists the genes included in each genetic test. Determinations of the pathogenicity of gene alterations, including pathogenic, likely pathogenic, variant of uncertain significance (VUS), and benign, were considered based on each company’s report and the ClinVar database.

Genetic counseling was provided before and after multigene panel testing. During the pre-test genetic counseling, medical and genetic information was collected, including the patient's medical history, family history, and results of previous tests. Information about possible familial tumors was then provided, as well as information to help the patient select a multigene panel test that met their needs. The potential benefits and disadvantages of multigene panel testing were discussed with the client during the pre-test counseling. If the patient was found to have a hereditary tumor based on the test results, post-test genetic counseling provided a surveillance plan, information on how to deal with blood relatives, and information on available social resources, and counseled the patient about what to do in the future.

## Results

During the study period, 36 individuals (all women) underwent multigene panel testing for suspected hereditary breast cancer, 30 of whom had a history of breast cancer and 6 of whom had no history of breast cancer but had a family history of breast cancer. Comprehensive genomic profiling for recurrent breast cancer was conducted in 3 of the 36 patients who were suspected of having hereditary breast cancer as a secondary finding. The remaining 33 women were suspected of having hereditary breast cancer based on their history and/or their family history of breast cancer. At the time of genetic testing, the median age of the 36 women was 47 years (range, 24–70 years), the median age of the 30 who had breast cancer was 47 years (range, 33–70 years), and the median age of the 6 who had no history of breast cancer was 52 years (range, 24–70 years). The median age of cancer onset in the 30 patients with a history of breast cancer was 41 years (range, 29–57 years). Table 1 summarizes the background information of the 36 individuals who underwent multigene panel testing, including the histopathological diagnoses of the 30 breast cancers.

**Table 1:** Backgrounds of the 36 women who underwent multigene panel testing

	Development of breast cancer	
	Present	Absent
No. of individuals	30	6
Age at the time of genetic testing, median (range), y	47 (33–70)	52 (24–70)
Age at the onset of breast cancer, median (range), y	41 (29–57)	NA
Family history of breast cancer		
Absent	11	0
Present	19	6
Type of breast cancer		
IDC, luminal	17	NA
IDC, luminal HER2	1	NA
IDC, HER2	1	NA
IDC, triple-negative	5	NA
DCIS	4	NA
Unknown	2	NA

*IDC* invasive ductal carcinoma, *HER2* human epidermal growth factor receptor 2, *DCIS* ductal carcinoma in situ, *NA* not applicable

**Fig. 1:**
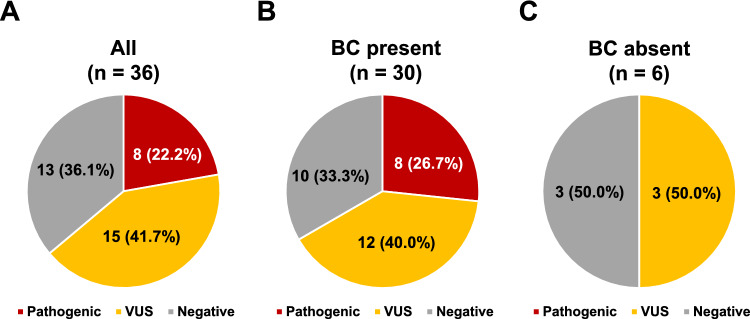
Pie charts showing the results of multigene panel testing of the 36 individuals in this study. **A** Results of all 36 individuals. **B** Results of the 30 individuals who had breast cancer (**BC**). **C** Results of six individuals who had no BC history. *VUS,* variant of uncertain significance

Utilizing multigene panel testing, at least one alteration was found in 23 of the 36 patients, and a total of 37 variants were found in these 23. Eight of the 36 (22.2%) had a pathogenic/likely pathogenic variant, with or without other VUSs, 15 (41.7%) had VUSs only, and 13 (36.1%) had negative genetic test results (Fig. 1). Eight of the 30 patients who had a history of breast cancer (26.7%) had a pathogenic/likely pathogenic variant with or without other VUSs, 12 (40.0%) had VUSs only, and 10 (33.3%) had negative genetic test results. None of the six women without any history of breast cancer had a pathogenic/likely pathogenic variant, three (50%) had VUSs only, and three (50%) had negative genetic test results (Fig. 1).

**Fig. 2:**
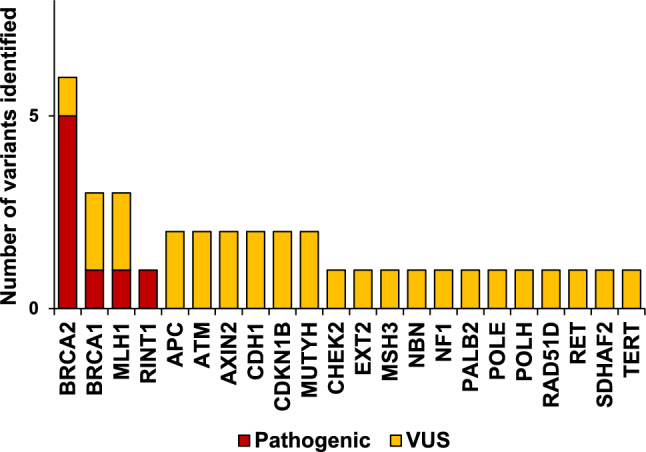
Frequency of variants detected by multigene panel testing in the 36 individuals. Red indicates a pathogenic variant, and yellow indicates a variant of uncertain significance (VUS)

Figure. 2 shows a breakdown of the 37 variants, including 8 pathogenic variants (21.6%) and 29 VUSs (78.4%), identified in 22 genes in this study. Among the eight pathogenic variants, six were variants in either *BRCA1* (*n* = 1) or *BRCA2* (*n* = 5), while one was a *MLH1* pathogenic variant and one was a *RINT1* pathogenic variant (Fig. 2 and Table 2). VUSs included *BRCA1* and *BRCA2*, as well as other breast cancer-associated genes, such as *ATM* (*n* = 2), *CDH1* (*n* = 2), *CHEK2* (*n* = 1), *NF1* (*n* = 1), *NBN* (*n* = 1), *PALB2* (*n* = 1), and *RAD51D* (*n* = 1; Fig. 2 and Tables 2, 3). The VUSs also included other cancer syndrome-related genes, such as *MLH1* (*n* = 2), *MUTYH* (*n* = 2), *APC* (*n* = 2), and *RET* (*n* = 1; Fig. 2 and Tables 2, 3).

**Table 2:** Eight individuals with pathogenic/likely pathogenic variants

No.	Gene	Variants	Interpretation at time of genetic testing	Interpretation by ClinVar database at 2024/09	Breast cancer
1	*BRCA1*	c.3122C>G, p.Ser1041Ter	Pathogenic	Pathogenic	TN
2	*BRCA2*	c.5645C>A, p.Ser1882Ter	Pathogenic	Pathogenic	Luminal
3	*BRCA2*	c.5645C>A, p.Ser1882Ter	Pathogenic	Pathogenic	DCIS
4	*BRCA2*	c.6952C>T, p.Arg2318Ter	Pathogenic	Pathogenic	Luminal
5	*BRCA2*	c.6768T>A, p.Cys2256*	Pathogenic	Pathogenic	Luminal
	*NTHL1*	c.637G>A, p.Ala213Thr	VUS	No data	
6	*BRCA2*	c.5645C>A, p.Ser1882Ter	Pathogenic	Pathogenic	Luminal
	*APC*	c.30476T>C	VUS	No data	
	*POLE*	c.2209A>G, p.Thr737Ala	VUS	VUS	
	*SDHAF2*	c.330C>A, p.Asn110Lys	VUS	VUS	
7	*MLH1*	c.306+1G>A	Pathogenic	Pathogenic	TN
8	*RINT1*	c.1603C>T, p.Arg535*	Pathogenic	Pathogenic	HER2

*TN* triple-negative, *DCIS* ductal carcinoma in situ, *VUS* variant of uncertain significance, *HER2* human epidermal growth factor receptor 2

**Table 3:** Fifteen individuals with variants of uncertain significance

No.	Gene	Variants	Interpretation at time of genetic testing	Interpretation by ClinVar database at 2024/09	Breast cancer
9	*APC*	c.−30476T>C	VUS	VUS	ND
10	*ATM*	c.2804C>G, p.Thr935Arg	VUS	Conflicting classifications of pathogenicity	Luminal
11	*ATM*	c.1741T>G, p.Leu581Val	VUS	VUS	TN
12	*AXIN2*	c.1498C>T, p.Leu500Phe	VUS	VUS	ND
13	*BRCA1*	c.5099C>T, p.Thr1700Ile	VUS	Likely pathogenic	TN
14	*BRCA1*	c.1879G>A, p.Val627Ile	VUS	Conflicting classifications of pathogenicity	ND
15	*BRCA2*	c.2350A>G, p.Met784Val	VUS	Benign	Luminal
16	*CDH1*	c.1018A>G, p.Thr340Ala	VUS	Benign	Luminal
17	*CDKN1B*	c.443G>T, p.Cys148Phe	VUS	Conflicting classifications of pathogenicity	Luminal
	*MUTYH*	c.493G>A, p.Ala165Thr	VUS	VUS	
18	*CHEK2*	c.1561C>T, p.Arg521Trp	VUS	VUS	DCIS
19	*MUTYH*	c.842C>T, p.Ala281Val	VUS	VUS	Luminal
	*PALB2*	c.1684+11A>G	VUS	Conflicting classifications of pathogenicity	
	*TERT*	c.2177C>T, p.Thr726Met	VUS	Conflicting classifications of pathogenicity	
20	*NBN*	c.1516C>G, p.Gln506Glu	VUS	VUS	Luminal
21	*POLH*	c.1766A>C, p.Lys589Thr	VUS	VUS	ND
22	*RAD51D*	c.80C>A, p.Thr27Lys	VUS	Conflicting classifications of pathogenicity	Luminal
23	*RET*	c.341G>A, p.Arg114His	VUS	Benign	Luminal
	*EXT2*	c.365C>T, p.Thr122Met	VUS	VUS	
	*CDH1*	c.1612G>A, p.Asp538Asn	VUS	VUS	
	*AXIN2*	c.1987T>G, p.Trp663Gly	VUS	Conflicting classifications of pathogenicity	
	*MLH1*	c.649C>T, p.Arg217Cys	VUS	Conflicting classifications of pathogenicity	

*VUS* variant of uncertain significance, *ND* not described, *TN* triple-negative, *DCIS* ductal carcinoma in situ

**Fig. 3:**
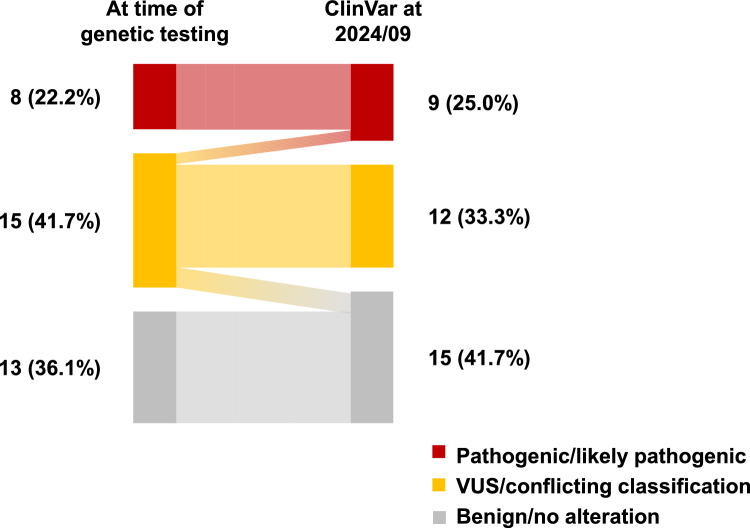
Changes in the interpretation of variants between the test report at the time of genetic testing and the ClinVar database for 2024/09. *VUS,* variant of uncertain significance

In compiling the data, we reassessed the pathogenic/VUS/benign status of each gene variant against the ClinVar database as of September, 2024. Interestingly, we found that one of the variants (*BRCA1*, c.5099C>T, p.Thr1700Ile) identified as a VUS at diagnosis was re-determined as likely pathogenic, and another three variants (*BRCA2*, c.2350A>G, p.Met784Val; *CDH1*, c.1018A>G, p.Thr340Ala; and *RET*, c.341G>A, p.Arg114His) identified as VUSs at diagnosis were re-determined as benign in the latest ClinVar database (Tables 2, 3). Consequently, the number of individuals with VUSs decreased from 15 (41.7%) at the time of multigene panel testing to 12 (33.3%) at the time of writing (Fig. 3).

## Discussion

NGS-based germline multigene panel testing is an efficient way to examine multiple genes involved in hereditary cancers at once, and it is also useful for patients suspected of having hereditary breast cancer [21]. While germline testing for *BRCA1/2* is covered by National Health Insurance in Japan, multigene panel testing is fully self-funded. Thus, it is not performed widely because of the high cost and because the follow-up system after testing is not yet well organized [19, 20]. The current study summarized the current status of multigene panel testing at our institution. VUSs are often identified in multigene panel testing and it is important to follow-up any individuals who have VUSs, keeping in mind that the evaluation of a VUS can change, as demonstrated in this study.

Of the 37 variants found in the multigene panel test in this study, 29 (78.4%) were VUSs. Among the VUSs identified, many were in genes known to cause hereditary breast cancer, such as *ATM*, *CDH1*, *CHEK2*, *PALB2*, and *RAD51D*, in addition to *BRCA1/2*, as well as in genes responsible for other hereditary tumor syndromes, such as *APC*, *MLH1*, *MSH3*, and *RET*. Importantly, it should be noted with extreme caution that some of the VUS evaluations changed between the time of diagnosis and the present [22]. The *BRCA1/2* gene is one of the most commonly tested genes, and the significance of many of the variants is considered to be confirmed because of the large amount of data available. However, in one of our study patients, with triple-negative breast cancer and a family history, the *BRCA1* variant (c.5099C>T, p.Thr1700Ile) had changed from a VUS at the time of testing to likely pathogenic in the latest ClinVar database. Moreover, three of the genes that were previously identified as VUSs (*BRCA2*, c.2350A>G, p.Met784Val; *CDH1*, c.1018A>G, p.Thr340Ala; and *RET*, c.341G>A, p.Arg114His) are now determined by ClinVar to be benign. Thus, it is important to monitor individuals with VUSs, since a VUS can be classified as pathogenic or benign with the accumulation of data.

In terms of the percentage of individuals in this study who had a VUS, 15 of the 36 (41.7%) had a VUS only and no pathogenic variant. Previous reports have reported a high frequency of VUS in multigene panel testing, generally exceeding 40% [23–26]. As in our case, VUS is more likely to turn benign than pathogenic [27], so VUS should not be used to guide medical management, nor should treatment proceed based on VUS [28]. Taken together, a decision must not be made until the clinical significance of the VUS is known, and since the multigene panel testing will find a VUS in many individuals, many subjects will need ongoing follow-up.

Of the 36 individuals tested in this study, 8 (22%) were found to have pathogenic variants, which represents a high frequency. This is partly because we included individuals from 2016 to 2019, before *BRCA1/2* genetic testing was covered by National Health Insurance. During that period, individuals with a strong suspicion of heredity were being offered multigene panel testing instead of *BRCA1/2*-only germline tests, because of the high cost of both *BRCA1/2*-only and multigene panel testing. Consequently, the frequency of pathogenic variants of *BRCA1/2* in this study was also high, (*n* = 5; 13.9%), compared with the commonly reported frequency of *BRCA1/2* variants in breast cancer, which is 4%–5% [5].

In addition to *BRCA1/2*, we found variants in *MLH1* and *RNT1* among the pathogenic variants identified in this study. A patient with a variant in *MLH1* identified is under surveillance for Lynch syndrome. *RINT1* does not represent a moderate-penetrance breast cancer susceptibility gene [29], so surveillance was deemed unnecessary for an individual with a variant in *RINT1*. However, biallelic variants in *RINT1* have been reported to cause liver failure and other problems [30, 31]; therefore, they could be considered an unaffected carrier, so genetic counseling was offered to provide this information. Taken together, each pathogenic variant identified by multigene panel testing requires a unique follow-up system and individualized care for every variant, since each gene has different penetrance, different organs susceptible to cancer, and in some cases may be associated with diseases other than cancer.

In Japan, National Health Insurance does not yet cover multigene panel testing of the germline, meaning patients must pay the full cost. If testing was covered by National Health Insurance, patients would have to pay only 10% to 30% of the cost, depending on their age. Moreover, all medical procedures based on test results not covered by National Health Insurance are not covered by this insurance and must be fully funded by the patient. For instance, if prophylactic resection or surveillance is required based on the results of multigene panel testing, the additional cost to the patient will be high. The benefit of performing multigene panel testing is that it improves the detection rate of genetic variants and increases the likelihood that a patient will be diagnosed with a hereditary tumor [19, 20]. The harm of testing is that if a variant is identified, many gene-based risk measures for familial tumors have not been established yet, and VUSs are also identified at high rates, which can lead to unnecessary anxiety and the disadvantages of excessive testing and treatment [19, 20].

This study has some limitations. First, it was retrospective in nature with a small number of samples; however, it enabled us to identify trends in germline variants in patients suspected of having hereditary breast cancer and demonstrate the features of multigene panel testing and points to be aware of. Second, the identification of germline variants by multigene panel testing is not yet widespread in Japan and there are many challenges for performing multigene panel testing, such as: (i) the cost of the test, which is not covered by National Health Insurance; (ii) the fact that surveillance and prophylactic resection based on the results of the gene test are at the patient’s own expense, and an adequate follow-up system after the test has not been established; (iii) the fact that VUSs are common and such patients require ongoing follow-up; and (iv) the gene panels covered differ depending on the testing laboratory, and are not standardized as they are subject to additions and deletions of genes at any time.

In conclusion, we reported on the current status of multigene panel testing performed for individuals with suspicious hereditary breast cancer in our institute. Our results demonstrate that multigene panel testing is a useful tool that can identify variants. One of the outcomes of this testing is that VUSs are frequently identified, and it is important to monitor these individuals because VUS evaluations can change over time with the accumulation of data. In Japan, the experience in genetic medicine that has been cultivated for hereditary breast and ovarian cancer treatment should be applied and utilized for patients with genetic variants other than *BRCA1/2*.

## Supplementary Information

Below is the link to the electronic supplementary material.

Supplementary file1 (DOCX 16 KB)
