# Supplementary material for: Germline variants detected by multigene panel testing in patients with suspected hereditary breast cancer
Source: Surg Today. 2025 Jan 20;55(8):1061–7. doi: 10.1007/s00595-025-02994-3 (PMC12339588; doi:10.1007/s00595-025-02994-3)
Supplement: Supplementary file 1 — Supplementary file1 (DOCX 16 KB) [file 595_2025_2994_MOESM1_ESM.docx]

| **Supplementary Table 1.** List of multiple gene tests | | | |
| --- | --- | --- | --- |
| Multigene panel test | Company | Number of genes | Gene list |
| Invitae Multi-Cancer Panel | Invitae,  San Francisco, CA, USA | 84 genes | AIP, ALK, APC, ATM, AXIN2, BAP1, BARD1, BLM, BMPR1A, BRCA1, BRCA2, BRIP1, CASR, CDC73, CDH1, CDK4, CDKN1B, CDKN1C, CDKN2A, CEBPA, CHEK2, CTNNA1, DICER1, DIS3L2, EGFR, EPCAM, FH, FLCN, GATA2, GPC3, GREM1, HOXB13, HRAS, KIT, MAX, MEN1, MET, MITF, MLH1, MSH2, MSH3, MSH6, MUTYH, NBN, NF1, NF2, NTHL1, PALB2, PDGFRA, PHOX2B, PMS2, POLD1, POLE, POT1, PRKAR1A, PTCH1, PTEN, RAD50, RAD51C, RAD51D, RB1, RECQL4, RET, RUNX1, SDHA, SDHAF2, SDHB, SDHC, SDHD, SMAD4, SMARCA4, SMARCB1, SMARCE1, STK11, SUFU, TERC, TERT, TMEM127, TP53, TSC1, TSC2, VHL, WRN, WT1 |
| Sentis Hereditary Cancer Panel  for women | BGI,  Cambridge, MA, USA | 74 genes | ALK, APC, ATM, AXIN2, BAP1, BARD1, BLM, BMPR1A, BRCA1, BRCA2, BR/Pl, CDC73, CDH1, CDK12, CDK4, CDKN18, CDKN2A, CHEK2, EPCAM, EXT1, EXT2, FANCG, FH, FLCN, GALNT12, KIT, MAX, MEN1, MET, MLH1, MLH3, MRE11A, MSH2, MSH3, MSH6, MUTYH, NBN, NF1, NF2, NTHL1, NTRK1, PALB2, PDGFRA, PHOX28, PMS1, PMS2, POLD1, POLE, PRSS1, PTCH1, PTCH2, PTEN, RADSO, RAD51C, RAD51D, RB1, RET, SOHA, SOHAF2, SOHB, SOHC, SOHO, SMA04, SMARCA4, SPINK1, STK11, SUFU, TMEM127, TP53, TSC1, TSC2, VHL, WT1, XPC |
| Sentis Hereditary Breast and Ovarian Cancer | BGI,  Cambridge, MA, USA | 26 genes | BRCA1, BRCA2, CHEK2, PALB2, BR/Pl, TP53, PTEN, STK11, CDH1, ATM, BARD1, MLH1, MRE11A, MSH2, MSH6, MUTYH, NBN, PMS1, PMS2, RADSO, RAD51C, RAD51D,  NF1, EPCAM, SMARCA4, CDK12 |
| Comprehensive Hereditary Cancer Panel Plus | Blueprint Genetics, Espoo, Finland | 160 genes | AIP, ALK, ANKRD26, APC, ATM, AXIN2, BAP1, BARD1, BLM, BMPR1A, BRAF, BRCA1, BRCA2, BRIP1, BUB1B, CBL, CD70, CDC73, CDH1, CDK4, CDKN1B, CDKN1C, CDKN2A, CEBPA, CEP57, CHEK2, CTNNA1, CYLD, DDB2, DDX41, DICER1, DIS3L2, DKC1, EFL1, EGFR, ELANE, EPCAM, ERCC1, ERCC2, ERCC3, ERCC4, ERCC5, ETV6, EXO1, EXT1, EXT2, EZH2, FAM111B, FANCA, FANCB, FANCC, FANCD2, FANCE, FANCF, FANCG, FANCI, FANCL, FANCM, FH, FLCN, GALNT12, GATA2, GPC3, GPR101, GREM1, HAVCR2, HNF1A, HOXB13, HRAS, IKZF1, KIF1B, KIT, KITLG, KRAS, LZTR1, MAP2K1, MAP2K2, MAX, MEN1, MET, MITF, MLH1, MLH3, MRE11A, MSH2, MSH3, MSH6, MUTYH, NBN, NF1, NF2, NRAS, NSD1, NSUN2, NTHL1, PALB2, PAX5, PDGFRA,, PHOX2B, PMS1,, PMS2, POLD1, POLE, POLH, POT1, PPM1D, PRF1, PRKAR1A, PTCH1, PTEN, PTPN11, RAD50, RAD51C, RAD51D, RAF1, RASA2, RB1, RECQL, RECQL4, REST, RET, RHBDF2, RIT1, RPS20, RRAS, RUNX1, SAMD9, SAMD9L, SBDS, SDHA, SDHAF2, SDHB, SDHC, SDHD,, SHOC2, SLX4, SMAD4, SMARCA4, SMARCB1, SMARCE1, SOS1, SOS2, SPRED1, SRP72, STK11, SUFU, TERC, TERT, TINF2, TMEM127, TP53, TRIP13, TSC1, TSC2, VHL, WRN, WT1, XPA, XPC and XRCC2. |
|  |  |  |  |
